# Examining interactions between polygenic scores and interpersonal trauma exposure on alcohol consumption and use disorder in an ancestrally diverse college cohort

**DOI:** 10.3389/fgene.2023.1274381

**Published:** 2024-02-01

**Authors:** Christina M. Sheerin, Rowan K. O’Hara-Payne, Eva E. Lancaster, Hailie Suarez-Rivas, Spit for Science Working Group, Fazil Aliev, Amy E. Adkins, Ananda Amstadter, Thomas Bannard, Peter Barr, Erin C. Berenz, Katie Bountress, Holly Byers, A. Christian Pais, Erin Caraway, James S. Clifford, Megan Cooke, Karen Chartier, Seung B. Cho, Elizabeth Do, Danielle M. Dick, Alexis C. Edwards, Renolda Gelzinis, Neeru Goyal, Sage Hawn, Laura M. Hack, Lisa J. Halberstadt, Sally Kuo, Jacquelyn L. Meyers, Emily Lasko, Jennifer Lend, Emily Lilley, Mackenzie Lind, Elizabeth Long, Alexandra Martelli, Arden Moscati, Anne Morris, Ashlee Moore, Kerry Mitchell, Aashir Nasim, Zoe Neale, Jill Opalesky, Cassie Overstreet, Kimberly Pedersen, Roseann E. Peterson, Tarah Raldiris, Brien Riley, Jessica Salvatore, Jeanne Savage, David Sosnowski, Rebecca Smith, Jinni Su, Cuie Sun, Nathaniel Thomas, Chloe Walker, Marcie Walsh, Bradley T. Webb, Teresa Willoughby, Brandon Wormley, Madison Woodroof, Jia Yan, Chris Chatzinakos, Elizabeth C. Prom-Wormley, Roseann E. Peterson

**Affiliations:** ^1^ Virginia Institute for Psychiatric and Behavioral Genetics, Virginia Commonwealth University, Richmond, VA, United States; ^2^ Department of Psychiatry, Virginia Commonwealth University, Richmond, VA, United States; ^3^ Center for Biological Data Science, Virginia Commonwealth University, Richmond, VA, United States; ^4^ Department of Psychology, Virginia Commonwealth University, Richmond, VA, United States; ^5^ Department of Psychiatry and Behavioral Sciences, SUNY Downstate Health Sciences University, Brooklyn, NY, United States; ^6^ Institute for Genomics in Health, SUNY Downstate Health Sciences University, Brooklyn, NY, United States; ^7^ Department of Epidemiology, Virginia Commonwealth University, Richmond, VA, United States

**Keywords:** alcohol consumption, alcohol use disorder (AUD), interpersonal trauma, polygenic score (PGS), college and university students, physical assault, sexual assault, trans-ancestry

## Abstract

**Introduction:** Genetic factors impact alcohol consumption and use disorder (AUD), with large-scale genome-wide association studies (GWAS) identifying numerous associated variants. Aggregate genetic methods in combination with important environmental factors (e.g., interpersonal trauma [IPT]) can be applied to expand our understanding of the ways by which genetic and environmental variables work together to influence alcohol consumption and disordered use. The present study aimed to detail the relationships between genome-wide polygenic scores (PGS) for alcohol phenotypes (i.e., alcohol consumption and AUD status) and IPT exposure as well as the interaction between them across ancestry.

**Methods:** Data were drawn from the Spit for Science (S4S) study, a US college student population, where participants reported on IPT exposure prior to college and alcohol consumption and problems during college (N = 9,006; ancestry: 21.3% African [AFR], 12.5% Admixed Americas [AMR], 9.6% East Asian [EAS], 48.1% European [EUR], 8.6% South Asian [SAS]). Two trans-ancestry PGS were constructed, one for alcohol consumption and another for AUD, using large-scale GWAS summary statistics from multiple ancestries weighted using PRS-CSx. Regression models were applied to test for the presence of associations between alcohol-PGS and IPT main and interaction effects.

**Results:** In the meta-analysis across ancestry groups, IPT exposure and PGS were significantly associated with alcohol consumption (β_*IPT*_ = 0.31, *P*_*IPT*_ = 0.0002; β_*PGS*_ = 0.09, *P*_*PGS*_ = 0.004) and AUD (OR_*IPT*_ = 1.12, *P*_*IPT*_ = 3.5 × 10^−8^; OR_*PGS*_ = 1.02, *P*_*PGS*_ = 0.002). No statistically significant interactions were detected between IPT and sex nor between IPT and PGS. When inspecting ancestry specific results, the alcohol consumption-PGS and AUD-PGS were only statistically significant in the EUR ancestry group (β_*PGS*_ = 0.09, *P*_*PGS*_ = 0.04; OR_*PGS*_ = 1.02, *P*_*PGS*_ = 0.022, respectively).

**Discussion:** IPT exposure prior to college was strongly associated with alcohol outcomes in this college-age sample, which could be used as a preventative measure to identify students at high risk for problematic alcohol use. Additionally, results add to developing evidence of polygenic score association in meta-analyzed samples, highlighting the importance of continued efforts to increase ancestral representation in genetic studies and inclusive analytic approaches to increase the generalizability of results from genetic association studies.

## Introduction

Alcohol use disorder (AUD) is common, with a lifetime prevalence of 29.1% in US adults (Grant et al., 2015), and is a significant public health concern (Sacks et al., 2015). Although national surveys of adolescents and young adults have demonstrated trends towards lower alcohol use overall, increases in high intensity drinking have been demonstrated (Hingson et al., 2017; Miech et al., 2023). This developmental period represents a time of increased risk for problematic alcohol use and the development of symptoms of AUD that contribute to patterns of use and development of AUD later in life (Prince et al., 2019). College students in particular are a high-risk population, with high rates of heavy drinking (Hingson et al., 2017; Ranker and Lipson, 2022). Compared to non-college, same-age peers, more college students engage in drinking (SAMHSA, 2021) and drink at higher amounts (Slutske et al., 2004; Slutske, 2005; Carter et al., 2010; Quinn and Kim, 2011). National epidemiologic surveys report that 30.7% of college students met criteria for AUD in 2012–2013, making it the most common mental health problem in college students (Arterberry et al., 2020). Problematic alcohol use in college is associated with immediate and long-term consequences on physical health (e.g., chronic physical disease (Scott et al., 2016)), emotional health (e.g., suicide risk (Hingson et al., 2009)), and functional outcomes (e.g., academic and job achievement (Williams et al., 2003)).

Twin and genetic association studies have demonstrated the importance of genetic factors on the development of alcohol phenotypes (e.g., consumption, disorder), with estimates of heritability ranging from ∼9% to 50% (Verhulst et al., 2015; Walters et al., 2018; Zhou et al., 2020). Further, large-scale genome-wide association studies (GWAS) have identified numerous significant single nucleotide polymorphisms (SNPs) for alcohol phenotypes including alcohol consumption (Clarke et al., 2017; Kranzler et al., 2019), problematic drinking (e.g., maximum habitual alcohol intake (Gelernter et al., 2019)); problematic alcohol use (PAU) defined as a combination of problem scores and alcohol use disorder (AUD) (Zhou et al., 2020); and alcohol dependence (AD) (Walters et al., 2018) and AUD diagnosis (Kranzler et al., 2019). While early work was conducted primarily in European ancestry (EUR) individuals (Peterson et al., 2019), more recent work has included additional ancestry populations and identified loci shared across populations (e.g., EUR and admixed African [AFR]) as well as ancestry-specific variants (Gelernter et al., 2019).

Alcohol phenotypes are highly polygenic, meaning that many variants of small effect size contribute to their development (Wray et al., 2014). Thus, aggregate genetic methods that capture cumulative common genetic risk for a given phenotype, via genome-wide polygenic scores (PGS), are increasingly utilized. PGS are an aggregated summation of genetic risk that, in general, are calculated for each individual by summing the number of risk variants they carry across the genome (Wray et al., 2021). Consumption and AUD PGS have been associated with alcohol-related disorders and DSM-IV alcohol dependence criterion counts (Walters et al., 2018; Kranzler et al., 2019). Consumption PGS have been associated with both higher frequency of alcohol consumption and increased dependence symptoms in young adults (Barr et al., 2019). Given the recognition that EUR-derived PGS, which are the most common given the over-representation in GWAS, decrease in performance across ancestries with increasing genetic distance, methods for trans-ancestry analyses are continuing to evolve and improve (Duncan et al., 2019). More recent work has demonstrated that cross-ancestry PGS perform better than single-ancestry PGS for alcohol (Zhou et al., 2023) and other physical health (Ge et al., 2022) phenotypes. Taken together, these findings highlight the continued need to increase sample sizes across the ancestry spectrum to realize the potential benefit of polygenic methods.

Environmental risk factors are also associated with increased prevalence of alcohol phenotypes. One important, established environmental risk factor for alcohol phenotypes is exposure to trauma, particularly interpersonal violence or trauma (IPT; i.e., physical or sexual assault or abuse). IPT has been associated with various forms of alcohol use (Charak et al., 2015), including increased alcohol consumption (Berenz et al., 2016), greater alcohol misuse (Kilpatrick et al., 2003), and increased AUD risk (Meyers et al., 2018). IPT is common, and rates are also high in individuals entering or in college, with 39% of incoming first year college students endorsing a history of IPT (Overstreet et al., 2017). In college students specifically, IPT has been associated with increased alcohol misuse (e.g. (Davis et al., 2002)). Compared with trauma exposure during later developmental periods (i.e., adulthood), evidence suggests that trauma during childhood/adolescence increases risk for alcohol misuse (Sartor et al., 2008).

Importantly, it is well established that environmental factors, such as IPT, may interact with genetic risk to substantially influence alcohol phenotypes (i.e., gene-environment interaction [GxE] (Dick and Kendler, 2012)). Indeed, there is growing evidence for the importance of the interaction between genetic and environmental factors on alcohol phenotypes (Prom-Wormley et al., 2017; Pasman et al., 2019). While much of the GxE work in alcohol phenotypes has focused on candidate genes or variants, more recent efforts have shifted to examination of PGS in line with our understanding of the polygenic nature of these traits (e.g., examining polygenic risk for alcohol misuse moderated by romantic partnerships (Barr et al., 2019)). Further, most previous research on GxE and alcohol phenotypes has primarily used populations of European descent (Chartier et al., 2017).

The primary objective of this project was to extend prior work by detailing the relationships between polygenic scores for alcohol phenotypes (i.e., alcohol consumption and AUD status) and IPT exposure. The impact of biological sex and ancestry on these relationships was also explored. It was hypothesized that 1) IPT exposure would be positively associated with alcohol consumption and AUD; 2) PGS for alcohol phenotypes would be significantly associated with alcohol consumption and AUD; 3) IPT exposure would moderate the association of PGS with alcohol consumption and AUD.

## Materials and methods

### Sample and procedures

Participants for the current study were included from an ongoing longitudinal cohort study of college students at a large, urban, mid-Atlantic public university. This study was approved by the university’s review board and all participants provided informed consent. For a detailed review of study methods see (Dick et al., 2014).

Briefly, incoming first-year students 18 years or older were invited to complete a baseline survey during their first (i.e., “freshman”) fall semester of college. Study data were collected and managed using REDCap electronic data capture tools (Harris et al., 2009; Harris et al., 2019) hosted at Virginia Commonwealth University. REDCap (Research Electronic Data Capture) is a secure, web-based application designed to support data capture for research studies. Survey items assessed demographics, personality and behavior, as well as family, friends, and experiences growing up, prior to entering college. Upon enrollment in the project and completion of the baseline survey, participants were invited to also provide a saliva sample for DNA analysis (98% of participants provided a sample). Participants who completed the baseline survey were invited again via email to complete subsequent longitudinal follow-up assessments each spring semester thereafter. These subsequent follow-up assessments asked questions regarding experiences since the prior assessment (i.e., past year). Individuals who did not participate in the first wave of data collection in the fall had the opportunity to join the study the following spring of their first year.

Data from five cohorts who matriculated in 2011, 2012, 2013, 2014, and 2017 were collected. Participants from these cohorts provided data during at least one of five points of data collection (N = 12,385). The sample reflected the self-reported racial and ethnic composition of the university population from which it was drawn: 47.9% White, 19.3% African-American, 16.6% Asian, 6.6% Hispanic/Latino, 9.6% other/multi-race/unknown or declined to respond. The current study included a subsample of participants with complete genotypic data and study variables (N = 9,006; Supplementary Figure S1).

### Study measures

#### Interpersonal trauma exposure

Interpersonal trauma exposure was measured using an abbreviated version of the Life Events Checklist (LEC (Gray et al., 2004)) for three items: experience of a physical assault, sexual assault, or other unwanted touching/sexual experience (the latter two sexual experience items were collapsed together), with a “yes” or “no” response. At the baseline time-point (i.e., year 1 fall), individuals reported on lifetime exposure experienced before attending college, to capture pre-college exposure. In the present study, these items were used to create a composite binary IPT exposure variable. A “yes” endorsement on any of the items represented “any” IPT type experienced prior to entering college.

#### Alcohol use variables

Two alcohol use variables were derived, reflecting alcohol consumption and AUD diagnosis. Participants who reported never drinking alcohol in their lifetime were not queried on further alcohol-related items and are thus not included in study analyses.

##### Consumption

Alcohol consumption was measured at each time point using the first two items from the Alcohol Use Disorders Identification Test (AUDIT (Bush et al., 1998)) that had participants report on their recent alcohol use with ordinal frequency and quantity items, asking 1) “how often do you have a drink containing alcohol?” and 2) “how many drinks containing alcohol do you have on a typical day when you are drinking?”. For each time point, these items were combined to create a single “grams of ethanol consumed per month” alcohol variable. These methods have been previously reported in prior work with this sample (Salvatore et al., 2016; Webb et al., 2017; Smith et al., 2021). Briefly, it involved first converting the categorical response options to the midpoints of the range for each option, then multiplying the product of these conversions by 14. Alcohol use was then natural-log transformed after adding a constant of one to adjust for positive skew and retain participants who consumed zero grams of alcohol (Salvatore et al., 2016; Webb et al., 2017). This resulted in a quantitative measure of average consumption at each assessment time point. In order to determine the impact of predictors on alcohol consumption, the highest of these scores across all time points was used for analyses, in order to identify the maximum habitual alcohol consumption. This approach was modeled after extant work (Gelernter et al., 2019), wherein the authors sought to reflect typical habitual maximum usage. This metric appears to have a stronger association with AUD risk as compared to maximum use ever, which may be a single occasion (Grant et al., 2009).

##### AUD diagnosis

Items related to DSM-5 (American Psychiatric Association, 2013) AUD were assessed using an adapted Semi-Structured Assessment for the Genetics of Alcoholism assessment (SSAGA (Bucholz et al., 1994)). Eleven past year DSM-5 symptoms (e.g., loss of control, craving, withdrawal symptoms) were assessed as 3-level Likert scale items (never, 1–2 times, 3 or more times) within each wave. An endorsement of 3 or more times on an item was considered a positive endorsement and coded as a 1. Negative endorsement or endorsement of 1–2 times on an item was coded as a 0. Responses to all items were used to create a sum symptom score. Participants who endorsed 2 or more symptoms were defined to be affected by AUD at each wave, resulting in a binary AUD variable. Participants missing 50% or more of these 11 items were coded as missing for that wave; otherwise, items were prorated (by averaging the endorsed items and multiplying by the total number of items in the scale) for missingness. Being affected by AUD at any of the waves was coded as being AUD affected for study analyses.

#### Biological sex

Biological sex was estimated from the genotypic data and used to assign each participant as either male (coded as 1) or female (coded as 2). Some participants were removed during the genotypic quality control steps due to indeterminate categorization by the PLINK software (Chang et al., 2015). Sex was included in these analyses because alcohol behaviors have been previously shown to differ by sex in prevalence and severity (Kandel et al., 1997; Nolen-Hoeksema, 2004; Erol and Karpyak, 2015).

### Genotyping and quality control

A detailed account of the primary cohort collection, genotyping methodology, and quality control procedures have been previously published (Dick et al., 2014; Peterson et al., 2017; Webb et al., 2017). DNA was extracted from saliva and genotyping was performed using three arrays (Affymetrix Axiom BioBank Array, SmokeScreen Genotyping Array, Infinium Global Screening Array-24 v3.0 BeadChip). For each of the three arrays, imputation and rigorous quality control procedures were performed (Webb et al., 2017) prior to association analyses. SHAPEIT2 (Delaneau et al., 2008) and IMPUTE2 (Howie et al., 2009) were used for imputation with the 1000 Genomes Project (1KGP) phase 3 reference panel (Sudmant et al., 2015; The 1000 Genomes Project Consortium et al., 2015).

#### Ancestry assignments and genetic principal components

Participants were assigned to one of five ancestral populations (either African [AFR], Admixed Americas [AMR], East Asian [EAS], European [EUR], or South Asian [SAS]) by selecting the minimum Mahalanobis distance between subjects and the 1KGP reference population via genetic-based principal component analysis (PCA) (Peterson et al., 2017). PCA was performed using EIGENSOFT SmartPCA (Patterson et al., 2006; Price et al., 2006). Additionally, within each ancestry the top ten principal components (PCs) were calculated from directly genotyped SNPs linkage disequilibrium pruned ranging 43–69 K depending on the ancestry (Peterson et al., 2017). These PCs were used as covariates in the regression models including PGS to reduce potential effects of population stratification.

#### Genome-wide polygenic scores (PGS)

As the genetic etiology of alcohol dependence is only partially shared with that of alcohol consumption (Polimanti et al., 2019), two alcohol-related PGS (one for consumption, one for disordered alcohol use) were constructed. PGS are an aggregated summation of genetic risk that, in general, are calculated for each individual by summing the number of risk variants they carry across the genome (Wray et al., 2021). Here, PGS were developed using PRS-CSx, a method that has been shown to perform well in large, diverse training samples by utilizing a Bayesian regression framework to adjust SNP effect sizes for local linkage disequilibrium patterns across populations (Ruan et al., 2022). PRS-CSx is able to combine GWAS results across ancestries, so that each alcohol-related PGS can be constructed using summary statistics from multiple large-scale GWAS. The alcohol consumption PGS combined summary statistics from the GWAS and Sequencing Consortium of Alcohol and Nicotine use (GSCAN; N_AFR_ = 8,078; N_AMR_ = 5,162; N_EAS_ = 90,852; N_EUR_ = 664,664, leaving out 23andMe & S4S data) (Saunders et al., 2022) and the Million Veterans Program (MVP; N_AFR_ = 56,495; N_EUR_ = 200,680) (Kranzler et al., 2019). The PGS predicting AUD was generated using the latest published results from MVP (EUR case N = 34,658; AFR case N = 17,267) (Kranzler et al., 2019). Since PRS-CSx requires a single set of GWAS summary statistics for each ancestry as input, the European and African ancestry alcohol consumption summary statistics were meta-analyzed by ancestry prior to analysis. Specifically, the European and African ancestry alcohol consumption summary statistics from GSCAN and MVP were meta-analyzed for each ancestry using inverse variance weighting (IVW) in METAL (Willer et al., 2010). The META weights from PRS-CSx were used to weight each SNP in the PGS and were constructed for each S4S participant using the profile option in PLINK (Purcell et al., 2007). There were approximately 250,000 SNPs included across the autosomes in each of the PGS. Prior to regression analyses, PGS were standardized to have a mean of 0 and a standard deviation of 1.

### Statistical analyses

Descriptive statistics and regressions were conducted using R (Version 4.3.1 (R Core Team, 2023)). Phenotypic-only models are presented in Supplementary Table S1. Regression models for each alcohol phenotype were constructed for each of the five ancestral groups in the S4S cohort. Additionally, covariates included ten ancestry-derived PCs in all models. The *lm* function from the stats package was used to conduct linear regression for the alcohol consumption models. The *blm* function from the blm package was used to conduct binary linear regression for the AUD models (Kovalchik and Varadhan, 2013). Binary linear regression models the probability of AUD endorsement as a linear function of explanatory predictor variables and has an added benefit of estimating model fit using R^2^. Consequently, an R^2^ was estimated for all models to determine the proportion of variance of the alcohol phenotype explained by the regression model. All models tested for statistical interactions on the additive scale. The full models that were tested are presented below (Equation 1; Equation 2). Regression results were then meta-analyzed across the five ancestry groups using METASOFT (Han and Eskin, 2011). The primary results were estimated using IVW fixed effect meta-analyses, which assumes that the effect sizes are similar across all groups. This assumption was examined by inspecting heterogeneity metrics via I^2^ and Cochran’s Q *p*-value. Since it is unknown if effects would vary by group, random effects meta-analyses, which allows for true effects to differ between groups, were also estimated. These results are presented in Supplementary Table S2.

Alcohol Consumption ∼ PCs+sex+IPT exposure+PGS+sex∗IPT exposure+PGS ∗ IPT exposure (1)

 AUD Diagnosis ∼PCs+sex+IPT exposure+PGS+sex ∗ IPT exposure+PGS ∗ IPT exposure (2)

## Results

Approximately 37.2% of the sample was exposed to IPT before attending college, demonstrating a high rate of trauma history in this sample. A greater proportion of female participants (40.6%) were exposed to IPT compared to males (31.3%).

**FIGURE 1:**
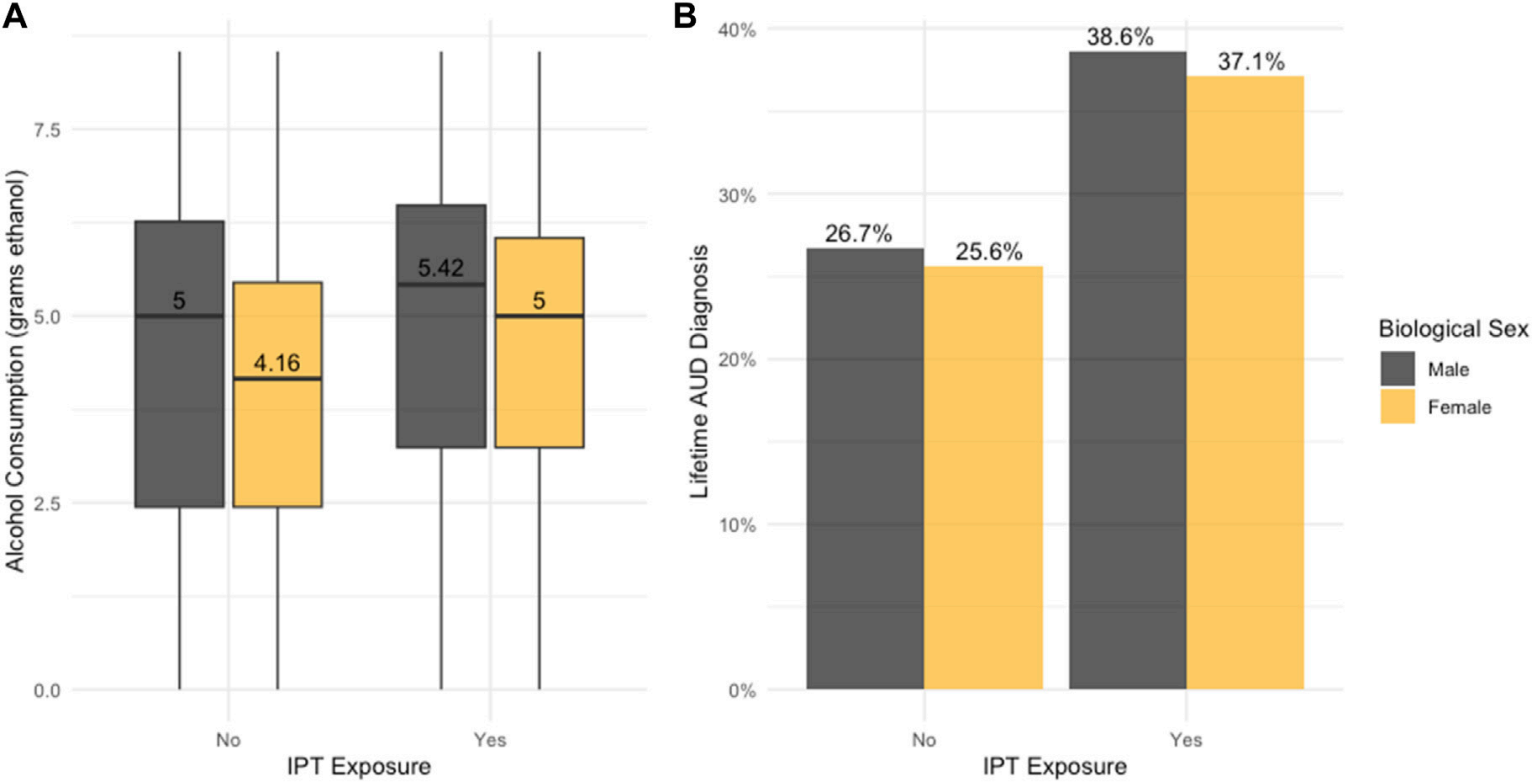
Sex Differences in IPT Exposure and Alcohol Phenotypes. **(A)** Boxplot illustrates sex differences in interpersonal trauma (IPT) and alcohol consumption (grams ethanol natural log transformed). **(B)** Barplot illustrates sex differences in the prevalence of IPT and alcohol use disorder (AUD).

As detailed in Table 1 (and visualized in Figure 1A), on average, participants across the study sample consumed a maximum average of 367 g of ethanol. Males consumed greater amounts of ethanol (490 g) than females (297 g; *p* = 1.07 × 10^−6^). Phenotypic-only analyses demonstrated that exposure to IPT (β = 0.385, *p* = 9.42 × 10^−6^) and sex were significantly associated with alcohol consumption (β = −0.377, *p* = 1.93 × 10^−9^). No statistically significant interaction between sex and IPT exposure on alcohol consumption was detected (Supplementary Table S1).

**TABLE 1:** Prevalence rates of interpersonal trauma and alcohol use behaviors by sex and genetic ancestry.

	Total	IPT exposure^a^	Lifetime AUD diagnosis^b^	Alcohol consumption mean (SD)^b^
Biological Sex				
Male	3,270 (*36.3%*)	1,023 (*31.3%*)	996 (*30%*)	490 (809)
Female	5,736 (63.7%)	2,331 (40.6%)	1737 (30.3%)	297 (516)
Genetic Ancestry				
African	1915 (*21.3%*)	700 (*36.5%*)	489 (*25.5%*)	255 (540)
Admixed Americas	1,126 (*12.5%*)	442 (*39%*)	364 (*32%*)	347 (*629*)
East Asian	862 (*9.6%*)	249 (*29%*)	222 (*25.8%*)	347 (*500*)
European	4,331 (*48.1%*)	1739 (*40.1%*)	1,444 (*33.3%*)	466 (715)
South Asian	772 (*8.6%*)	224 (*29%*)	214 (*27.7%*)	265 (*529*)
Total	9,006 (*100%*)	3,354 (*37.2%*)	2,733 (*30.3%*)	367 (644)

Note: IPT, interpersonal trauma; AUD, alcohol use disorder.

^a^ Variable refers to behavior prior to attending college.

^b^ Variable refers to behavior during college, mean alcohol consumption is in grams ethanol consumed per month.

In this sample, 30.3% of participants met study criteria for AUD diagnosis. The prevalence of AUD diagnosis did not significantly differ between females and males (Table 1; Figure 1B). Similarly, phenotypic-only analyses indicated that exposure to IPT was significantly associated with AUD (β = 0.544, *p* = 1.04 × 10^−11^), while sex was not (β = −0.059, *p* = 0.340). No significant statistical interaction was detected between sex and IPT exposure for AUD (Supplementary Table S1). A visual depiction of these findings is presented in Supplementary Figure S2 (forest plot of IPT effect on alcohol consumption and AUD) and Supplementary Figure S3 (forest plot of sex on alcohol consumption and AUD), for the combined meta-analysis sample and within-ancestry samples.

In the fixed-effects meta-analysis, including the test of the association of the PGS (Table 2), exposure to IPT was significantly associated with alcohol consumption and AUD (*p* = 2.13 × 10^−4^, *p* = 3.50 × 10^−8^, respectively) as hypothesized. On average, participants exposed to IPT consumed 3.72 g more alcohol (β = 0.313) and were 1.12 times more likely to meet AUD criteria during college (β = 0.110) compared to those who were not exposed. There were no significant statistical interactions between sex and IPT exposure on alcohol consumption or AUD. In line with study hypotheses, the alcohol-PGSs were significantly associated with alcohol consumption (β = 0.086, *p* = 3.97 × 10^−3^) and AUD (OR = 1.019, *p* = 1.17 × 10^−3^) but accounted for a small portion of the phenotypic variance (partial R^2^ range: 0.002%–0.39%; Supplementary Table S3). Contrary to hypotheses, there were no significant statistical interactions between either alcohol-PGS on alcohol consumption or AUD.

**TABLE 2:** Meta-Analysis of Regression Coefficients for Biological Sex, IPT, PGS, and Interactions Between them for Alcohol Use Behaviors Based on Fixed Effect Model.

Variable	*β*	Standard error	*p*-value	*I* ^2^	Cochran’s Q *p*-value
***Alcohol Consumption***					
**Sex**					
Male	Reference				
Female	**−0.300**	**0.061**	**1.02 x 10^−6^**	35.73	0.183
**IPT Exposure**					
No exposure	Reference				
IPT Exposure	**0.313**	**0.084**	**2.13 × 10** ^**–4**^	46.39	0.108
Sex by IPT Exposure	0.136	0.104	0.193	0.0008	0.407
**PGS**					
PGS	**0.086**	**0.030**	**3.97 x 10^−3^**	0.000	0.669
PGS by IPT Exposure	−0.047	0.049	0.333	0.000	0.795
***Alcohol Use Disorder***					
**Sex**					
Male	Reference				
Female	0.002	0.012	0.880	57.97	**0.049**
I**PT Exposure**					
No Exposure	Reference				
IPT Exposure	**0.110**	**0.020**	**3.50 x 10^−8^**	65.75	**0.020**
Sex by IPT Exposure	0.008	0.024	0.727	30.17	0.220
**PGS**					
PGS	**0.019**	**0.006**	**1.17 x 10^−3^**	0.000	0.878
PGS by IPT Exposure	−0.008	0.011	0.457	0.000	0.999

Note: IPT, interpersonal trauma; PGS, polygenic score, bolded estimates are significant at *p* < 0.05.

**FIGURE 2:**
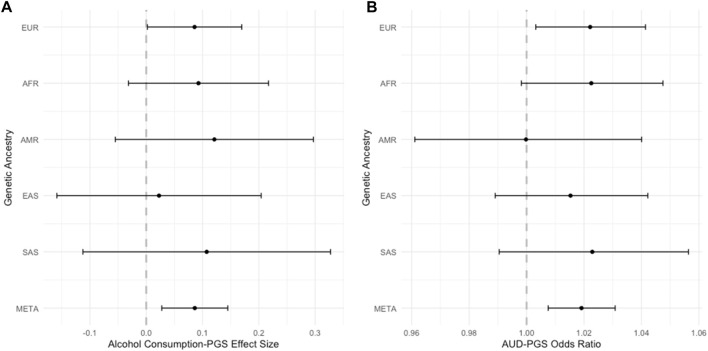
Effect of Alcohol-PGS on Alcohol Behaviors Across Ancestries. Forest plots showing the alcohol-PGS effect on **(A)** alcohol consumption and **(B)** alcohol use disorder (AUD) across ancestries and meta-analyzed (META).

The within-ancestry models are reported in Supplementary Table S3. The magnitude of associations varied across ancestry groups and alcohol outcome (alcohol consumption-PGS, β range*:* 0.023–0.121, *P* range*:* 0.806–0.045; AUD-PGS, β range*:* 0.0002–0.022, *P* range: 0.992–0.022). As shown in Figure 2, in the EUR ancestry sample the PGS were significantly associated with AUD and alcohol consumption. In the AFR ancestry sample, the PGS was nominally associated with AUD. In the AMR, EAS, and SAS ancestry samples, alcohol-PGS were not statistically significantly associated with AUD or consumption. No significant PGS interactions with IPT exposure or sex in the within-ancestry models were detected.

## Discussion

To our knowledge, this is the first study of young adults in a college setting that examines the association between IPT exposure and alcohol use behaviors while also accounting for the role of genetic factors through PGS as estimated using a meta-analysis across five ancestry groups. This study identified four primary conclusions. First, the association between sex and alcohol use behaviors was specific to maximum habitual alcohol consumption rather than AUD. Second, IPT exposure was associated with both alcohol consumption and AUD but there was limited evidence for the role of interactions between sex and IPT. Third, trans-ancestral meta-analyzed PGS were associated with both alcohol consumption and AUD. Fourth, there was limited evidence for the role of statistical interactions between PGS and IPT for alcohol behaviors.

### The role of sex on IPT and alcohol use behaviors

Rates of IPT exposure (37.2%) in this sample were high and in line with prior literature in a college age population (Cusack et al., 2019; Vrana and Dean, 1994; Slutske et al., 2004; Slutske, 2005; Blanco et al., 2008; Overstreet et al., 2017). Females reported higher rates of IPT compared to males (40.6%, 31.3%, respectively) as is well-established for interpersonal trauma, compared to accidental and other trauma exposures more broadly (Overstreet et al., 2017; Boyraz and Brandon Waits, 2018; Cusack et al., 2019). These findings highlight the at-risk nature of this developmental stage and environment, particularly for females. Rates of AUD status were also high (30.3%), also in line with prior literature (Cusack et al., 2019; Vrana and Dean, 1994; Slutske et al., 2004; Slutske, 2005; Blanco et al., 2008; Overstreet et al., 2017). Males reported greater alcohol consumption compared to females, as is generally expected (Nolen-Hoeksema, 2004; Erol and Karpyak, 2015). However, there were no differences in AUD prevalence by sex. This suggests that females are at similar risk than males for disordered alcohol outcomes in this college student sample. These findings are in line with more recent trends towards decreasing sex-related differences, particularly for disordered alcohol use (Harford et al., 2005; Grant et al., 2015; Hasin and Grant, 2015; Keyes et al., 2019).

### Significant associations between IPT and alcohol use behaviors

One aim of this study was to examine the main effects of IPT on alcohol consumption and AUD. As hypothesized, IPT exposure prior to entering college was positively associated with both alcohol consumption and AUD status, even when adjusting for sex, in support of an established literature (Berenz et al., 2016; Davis et al., 2002; Bountress et al., 2019). There was limited evidence for an interaction between IPT and sex on alcohol use behaviors. Some studies have found such an interaction (e.g., childhood maltreatment and sexual abuse significantly associated with alcohol problems in women but not men (Widom et al., 1995; Widom et al., 2006); IPT associated with greater baseline alcohol consumption in female college students, in the present study sample (Berenz et al., 2016)). However, other work in college samples has demonstrated that various types of IPT (e.g., intimate partner violence, childhood adversity) are associated with higher rates of alcohol use, greater risky drinking, and substance use equally for both genders (Coker et al., 2002; Simons et al., 2008; Grest et al., 2022). It may be that differences emerge with new-onset traumatic events (as found by Berenz and colleagues). It may also be that consideration of different measures of IPT is important, given different rates of physical assault and sexual assault by sex (Tolin and Foa, 2006; Basile et al., 2022; Thompson and Susannah, 2022) and potential for more nuanced associations in the relationship between assault type and outcomes (Meyers et al., 2018).

### Effect of PGS on alcohol use behaviors but no evidence for PGS by IPT interaction

This study also aimed to examine the main effects of alcohol-PGS and interaction effects with IPT exposure on maximum habitual alcohol consumption and AUD in a large, ancestrally diverse cohort. Alcohol consumption and AUD PGS were developed using PRS-CSx, to fully utilize the diverse training and target samples and to facilitate a cross-ancestry approach. Indeed, in the trans-ancestral meta-analysis, PGS were associated with both alcohol consumption and AUD status, as hypothesized. However, *post hoc* inspection of within-ancestry analyses demonstrated significant main effects of PGS on alcohol consumption and AUD only in the largest ancestry sample, EUR. A trend-level effect of PGS on AUD was also found in the second-largest AFR sample. There were no statistically significant associations of the PGS in the AMR, EAS, nor SAS samples, which were the smallest groups (N < 1,200). It is also noted that even the significantly associated PGSs accounted for only a small proportion of phenotypic variance (<0.5%). However, the effect sizes for the PGS were not significantly different by ancestry, although it is unknown whether this is simply due to wide confidence intervals from smaller sample sizes. The lack of interaction effects between either alcohol PGS and IPT, while contrary to study hypotheses, align with frequent lack of gene by environment effects in the literature, including within a subset of the present study sample (Su et al., 2018). Findings of a significant meta-analytic PGS association with both alcohol consumption and AUD support the use of methods designed for creating trans-ancestry PGS, rather than using single-ancestry PGS (Zhou et al., 2023), and extend prior work in this sample that did not find such associations using single-ancestry methods (Su et al., 2018; Ksinan et al., 2019). The lack of significant within-ancestry PGS findings for the AMR, EAS, and SAS samples, in part likely due to power, demonstrates the continued need to increase sample sizes across the ancestry spectrum to realize the potential benefits of polygenic prediction.

### Limitations and future directions

Despite strengths of the study including a large, ancestrally diverse sample, use of state-of-the-science methods for creating PGS with multiple ancestries, and preregistration of hypotheses and analyses on the Open Science Framework (https://doi.org/10.17605/OSF.IO/6875J), findings need to be interpreted in the light of existing limitations. First, many of the within-ancestry samples were still underpowered to detect genetic and interaction effects. Despite the improvements of using meta-analytic methods, limited diverse ancestry samples remains a general limitation of the alcohol and psychiatric genetics research field as a whole (Peterson et al., 2019). Efforts to expand inclusion of samples across the ancestry spectrum in genetic research remain essential. Second, all of our phenotypic measures were self-reported and thus may be subject to reporting bias, particularly given the nature of our retrospective measures focused on pre-college IPT and alcohol behaviors. Third, we focused on maximum habitual consumption and any AUD reported during college to highlight risk (genetic and pre-college IPT) on development of greatest alcohol-related problems during college. Stability or increases over time in alcohol consumption and disorder symptoms were not explicitly modeled, nor were the impact of new-onset college IPT events, which represents important next steps of this work. Finally, we used a dichotomous IPT exposure variable given limitations in the available survey data. However, further work in this area would benefit from measurement and examination of trauma severity, count, and event type given the likely further impact and nuances of each of these constructs.

As the landscape of genetic analytic methodologies and software are better equipped to incorporate samples across the ancestry spectrum our understanding of the genetic risk architecture of conditions will grow. For example, a number of alternative methods are available that can prioritize genes (e.g., fine mapping) such as transcriptome-wide association studies (TWAS (Chatzinakos et al., 2021)) and gene-based PGS are also being increasingly used in ancestrally diverse samples (Lai et al., 2022). Additionally, despite known large effects of environmental exposures on complex disease risk, like alcohol behaviors, there have been limited efforts to incorporate these factors into large-scale molecular genetic studies. Given the relative importance of environmental factors on alcohol use liability, there is a clear need to incorporate these factors into our etiological models going forward.

## Data Availability

The datasets presented in this article are not readily available because the NIH Genomic Data Sharing Policy states that data must be shared in a manner consistent with the research participants’ informed consent, and the confidentiality of the data and the privacy of participants must be protected. Requests to access the datasets should be directed to https://www.ncbi.nlm.nih.gov/projects/gap/cgi-bin/study.cgi?study_id=phs001754.v4.p2.

## The Spit for Science Working Group

Director: Karen Chartier, Co-Director: Ananda Amstadter. Past Founding Director: Danielle M. Dick. Registry management: Emily Lilley, Renolda Gelzinis, Anne Morris. Data cleaning and management: Katie Bountress, Amy E. Adkins, Nathaniel Thomas, Zoe Neale, Kimberly Pedersen, Thomas Bannard, and Seung B. Cho. Data collection: Kimberly Pedersen, Amy E. Adkins, Peter Barr, Holly Byers, Erin C. Berenz, Erin Caraway, Seung B. Cho, James S. Clifford, Megan Cooke, Elizabeth Do, Alexis C. Edwards, Neeru Goyal, Laura M. Hack, Lisa J. Halberstadt, Sage Hawn, Sally Kuo, Emily Lasko, Jennifer Lend, Mackenzie Lind, Elizabeth Long, Alexandra Martelli, Jacquelyn L. Meyers, Kerry Mitchell, Ashlee Moore, Arden Moscati, Aashir Nasim, Zoe Neale, Jill Opalesky, Cassie Overstreet, A. Christian Pais, Tarah Raldiris, Jessica Salvatore, Jeanne Savage, Rebecca Smith, David Sosnowski, Jinni Su, Nathaniel Thomas, Chloe Walker, Marcie Walsh, Teresa Willoughby, Madison Woodroof, and Jia Yan. Genotypic data processing and cleaning: Cuie Sun, Brandon Wormley, Brien Riley, Fazil Aliev, Roseann E. Peterson, and Bradley T. Webb.

## Supplementary material

SUPPLEMENTARY FIGURE S1 Study Sample Size. Flow chart showing sample sizes at each quality control step.

SUPPLEMENTARY FIGURE S2 Effect of IPT Exposure on Alcohol Behaviors Across Ancestries. Forest plots showing the interpersonal trauma (IPT) effect on **(A)** alcohol consumption and **(B)** alcohol use disorder (AUD) across ancestries and meta-analyzed (META).

SUPPLEMENTARY FIGURE S3 Effect of Biological Sex on Alcohol Behaviors Across Ancestries. Forest plots showing the biological sex effect on **(A)** alcohol consumption and **(B)** alcohol use disorder (AUD) across ancestries and meta-analyzed (META).
