# Supplementary material for: Examining interactions between polygenic scores and interpersonal trauma exposure on alcohol consumption and use disorder in an ancestrally diverse college cohort
Source: Front Genet. 2024 Feb 1;14:1274381. doi: 10.3389/fgene.2023.1274381 (PMC10868390; doi:10.3389/fgene.2023.1274381)
Supplement: Supplementary file 1 [file DataSheet1.pdf]

Supplemental Table 1. Phenotypic-only Regression Results for Biological Sex, IPT, and Interactions Between them for Alcohol Use Behaviors.

Variable	*β*	Standard Error	Odds Ratio [95% CI]	*p*-value
Alcohol Consumption				
**Sex**				
Male	Reference			
Female	**-0.377**	**0.063**	**0.686** [0.61, 0.78]	**1.93×10^{-9}**
**IPT Exposure**				
No exposure	Reference			
IPT Exposure	**0.385**	**0.087**	**1.470** [1.24, 1.74]	**9.42×10^{-6}**
Sex by IPT Exposure	0.137	0.107	1.147 [0.93, 1.41]	0.199
Alcohol Use Disorder				
**Sex**				
Male	Reference			
Female	-0.059	0.062	0.943 [0.84, 1.06]	0.340
**IPT Exposure**				
No exposure	Reference			
IPT Exposure	**0.544**	**0.080**	**1.722** [1.47, 2.01]	**1.04×10^{-11}**
Sex by IPT Exposure	-0.005	0.099	0.995 [0.82, 1.21]	0.959

Note: IPT = interpersonal trauma, bolded estimates are significant at p < 0.05.

Supplemental Table 2. Meta-Analysis of Regression Coefficients for Biological Sex, IPT, PGS, and Interactions Between them for Alcohol Use Behaviors Based on Random Effect Model.

Variable	β	Standard Error	*p*-value
Alcohol Consumption			
**Sex**			
Male	Reference		
Female	**-0.304**	**0.084**	**3.97×10^{-3}**
**IPT Exposure**			
No exposure	Reference		
IPT Exposure	**0.320**	**0.133**	**1.63×10^{-2}**
Sex by IPT Exposure	0.136	0.104	0.193
**PGS**			
PGS	**0.086**	**0.030**	**3.97×10^{-3}**
PGS by IPT Exposure	-0.047	0.049	0.333
Alcohol Use Disorder			
**Sex**			
Male	Reference		
Female	0.007	0.019	0.723
**IPT Exposure**			
No exposure	Reference		
IPT Exposure	**0.132**	**0.038**	**5.00×10^{-4}**
Sex by IPT Exposure	-0.003	0.031	0.919
**PGS**			
PGS	**0.019**	**0.006**	**1.16×10^{-3}**
PGS by IPT Exposure	-0.008	0.011	0.475

Note: IPT = interpersonal trauma, PGS = polygenic score, bolded estimates are significant at $p < 0.05$.

Supplemental Table 3. Regression Coefficients for Biological Sex, IPT, PGS, and Interactions Between them for Alcohol Use Behaviors Based on Fixed Effect Model Across Ancestries.

	**EUR** *N = 4331*	**AFR** *N = 1915*	**AMR** *N = 1126*	**EAS** *N = 862*	**SAS** *N = 772*	**META** *N = 9006*
Alcohol Consumption						
**PC1**						
β	0.188	-0.258	0.333	-0.385	0.223	0.044
Standard Error	0.034	0.051	0.087	0.079	0.092	0.024
p-value	$\mathbf{2.8\times10^{-8}}$	$\mathbf{4.1\times10^{-7}}$	$\mathbf{1.3\times10^{-4}}$	$\mathbf{1.4\times10^{-6}}$	**0.016**	0.074
**PC2**						
β	0.071	0.087	0.069	0.130	-0.070	0.070
Standard Error	0.033	0.051	0.070	0.079	0.092	0.024
p-value	**0.032**	0.086	0.328	0.099	0.443	**0.003**
**PC3**						
β	0.005	0.012	-0.068	0.229	0.071	0.023
Standard Error	0.034	0.051	0.069	0.078	0.093	0.024
p-value	0.892	0.809	0.324	**0.004**	0.448	0.338
**PC4**						
β	-0.024	0.005	0.275	0.104	-0.131	0.001
Standard Error	0.033	0.051	0.113	0.078	0.092	0.024
p-value	0.458	0.925	**0.015**	0.185	0.153	0.952
**PC5**						
β	0.074	-0.045	-0.020	-0.054	-0.096	0.014
Standard Error	0.034	0.051	0.077	0.078	0.092	0.024
p-value	**0.028**	0.374	0.789	0.492	0.295	0.560
**PC6**						
β	0.001	-0.006	0.214	-0.104	0.042	0.005
Standard Error	0.035	0.051	0.099	0.079	0.092	0.025
p-value	0.972	0.898	**0.031**	0.188	0.647	0.829
**PC7**						
β	0.018	0.015	-0.172	-0.069	-0.043	-0.008
Standard Error	0.035	0.051	0.095	0.081	0.093	0.025
p-value	0.607	0.765	0.070	0.400	0.644	0.735
**PC8**						
β	0.073	0.021	-0.082	0.082	0.005	0.037
Standard Error	0.037	0.051	0.072	0.078	0.092	0.025

p-value	0.052	0.679	0.257	0.295	0.953	0.141
**PC9**						
β	-0.011	0.023	-0.066	0.016	-0.038	-0.010
Standard Error	0.033	0.051	0.069	0.080	0.093	0.024
p-value	0.731	0.644	0.341	0.845	0.684	0.687
**PC10**						
β	0.014	-0.088	-0.041	0.083	0.055	-0.005
Standard Error	0.033	0.051	0.069	0.078	0.092	0.024
p-value	0.662	0.083	0.552	0.292	0.547	0.822
**Sex_{Female}**						
β	-0.280	-0.404	-0.408	0.087	-0.535	-0.300
Standard Error	0.086	0.141	0.179	0.191	0.220	0.061
p-value	**0.001**	**0.004**	**0.023**	0.649	**0.052**	**1.02×10^{-6}**
Partial R^2	0.004	0.004	0.008	0.002	0.008	
**IPT $Exposure_{Yes}$**						
β	0.347	-0.120	0.577	0.246	0.725	0.313
Standard Error	0.113	0.205	0.249	0.300	0.313	0.085
p-value	**0.002**	0.557	**0.021**	0.413	**0.021**	**2.13×10^{-4}**
Partial R^2	0.006	0.007	0.012	0.007	0.022	
**Sex_{Female} by IPT $Exposure_{Yes}$**						
β	0.019	0.523	-0.081	0.265	0.222	0.136
Standard Error	0.141	0.239	0.303	0.369	0.410	0.104
p-value	0.895	**0.029**	0.788	0.473	0.589	0.193
Partial R^2	3.9×10^{-6}	0.002	6.2×10^{-5}	5.8×10^{-4}	3.7×10^{-4}	
**$PGS_{consumption}$**						
β	0.086	0.093	0.121	0.023	0.107	0.086
Standard Error	0.043	0.063	0.090	0.092	0.112	0.030
p-value	**0.045**	0.144	0.178	0.806	0.338	**3.97×10^{-3}**
Partial R^2	0.001	0.001	0.002	7.8×10^{-4}	0.002	
**$PGS_{consumption}$ by IPT $Exposure_{Yes}$**						
β	-0.041	-0.146	-0.019	0.098	0.004	-0.047
Standard Error	0.067	0.106	0.140	0.176	0.201	0.049
p-value	0.544	0.166	0.895	0.578	0.982	0.333
Partial R^2	8.3×10^{-5}	0.001	1.5×10^{-5}	3.4×10^{-4}	6.3×10^{-7}	

Alcohol Use Disorder

**PC1**						
β	0.033	-0.029	0.001	-0.043	0.008	0.006
Standard Error	0.006	0.009	0.020	0.013	0.016	0.004
p-value	**8.9×10^{-8}**	**0.002**	0.947	**7.4×10^{-4}**	0.601	0.155
**PC2**						
β	0.006	-0.001	0.007	0.014	-0.017	0.003
Standard Error	0.007	0.009	0.016	0.013	0.017	0.004
p-value	0.410	0.885	0.668	0.276	0.295	0.481
**PC3**						
β	-0.008	0.010	7.4×10^{-4}	-0.005	-0.015	-0.003
Standard Error	0.008	0.011	0.016	0.016	0.016	0.005
p-value	0.320	0.334	0.964	0.778	0.362	0.574
**PC4**						
β	0.008	1.8×10^{-4}	0.018	0.021	0.008	0.008
Standard Error	0.006	0.009	0.026	0.016	0.018	0.005
p-value	0.186	0.984	0.490	0.188	0.648	0.104
**PC5**						
β	0.015	0.016	-0.017	-0.004	-0.003	0.005
Standard Error	0.005	0.011	0.015	0.005	0.018	0.003
p-value	**0.003**	0.156	0.282	0.444	0.861	0.137
**PC6**						
β	-0.017	-0.011	0.029	0.007	0.024	-0.001
Standard Error	0.009	0.010	0.023	0.007	0.018	0.004
p-value	0.046	0.295	0.198	0.334	0.186	0.776
**PC7**						
β	-0.002	-0.003	-0.013	0.001	0.002	-0.002
Standard Error	0.008	0.011	0.022	0.014	0.017	0.005
p-value	0.787	0.774	0.545	0.934	0.887	0.695
**PC8**						
β	0.010	-0.008	0.018	0.016	4.9×10^{-4}	0.006
Standard Error	0.008	0.010	0.017	0.014	0.017	0.005
p-value	0.213	0.453	0.277	0.268	0.977	0.237
**PC9**						
β	-0.002	0.007	0.011	0.022	0.004	0.009
Standard Error	0.007	0.011	0.016	0.007	0.016	0.004
p-value	0.799	0.536	0.508	**0.002**	0.811	**0.030**
**PC10**						
β	-0.004	0.001	0.005	-0.006	0.018	-0.001

Standard Error	0.008	0.010	0.016	0.010	0.020	0.005
p-value	0.600	0.888	0.737	0.563	0.370	0.832
**Sex_{Female}**						
β	-0.023	-0.002	-0.037	0.018	0.088	0.002
Standard Error	0.019	0.027	0.038	0.025	0.034	0.012
p-value	0.228	0.939	0.338	0.477	**0.01**	0.880
Partial R^2	9.3×10^{-5}	1.5×10^{-4}	0.002	4.7×10^{-5}	0.003	
**IPT $Exposure_{Yes}$**						
β	0.091	0.042	0.132	0.166	0.295	0.110
Standard Error	0.028	0.043	0.061	0.068	0.066	0.020
p-value	**1.26×10^{-3}**	0.322	**0.03**	**0.015**	**8.72×10^{-6}**	**3.50×10^{-8}**
Partial R^2	0.011	0.006	0.01	0.015	0.036	
**Sex_{Female} by IPT $Exposure_{Yes}$**						
β	0.037	0.047	-0.032	-0.054	-0.155	0.008
Standard Error	0.035	0.050	0.072	0.080	0.087	0.024
p-value	0.281	0.354	0.657	0.501	0.075	0.727
Partial R^2	2.66×10^{-4}	4.32×10^{-4}	1.77×10^{-4}	5.38×10^{-4}	4.63×10^{-3}	
**PGS_{AUD}**						
β	0.022	0.022	-0.0002	0.015	0.023	0.019
Standard Error	0.010	0.012	0.020	0.013	0.016	0.006
p-value	**2.17×10^{-2}**	0.070	0.992	0.256	0.169	**1.17×10^{-3}**
Partial R^2	0.001	0.002	1.5×10^{-5}	0.001	0.001	
**PGS_{AUD} by IPT $Exposure_{Yes}$**						
β	-0.007	-0.008	-0.005	-0.017	-0.007	-0.008
Standard Error	0.016	0.022	0.033	0.037	0.044	0.011
p-value	0.663	0.715	0.877	0.649	0.878	0.475
Partial R^2	4.4×10^{-5}	6.3×10^{-5}	2.7×10^{-5}	2.2×10^{-4}	7.6×10^{-5}	

Note: IPT = interpersonal trauma, PGS = polygenic score, bolded estimates are significant at p < 0.05.

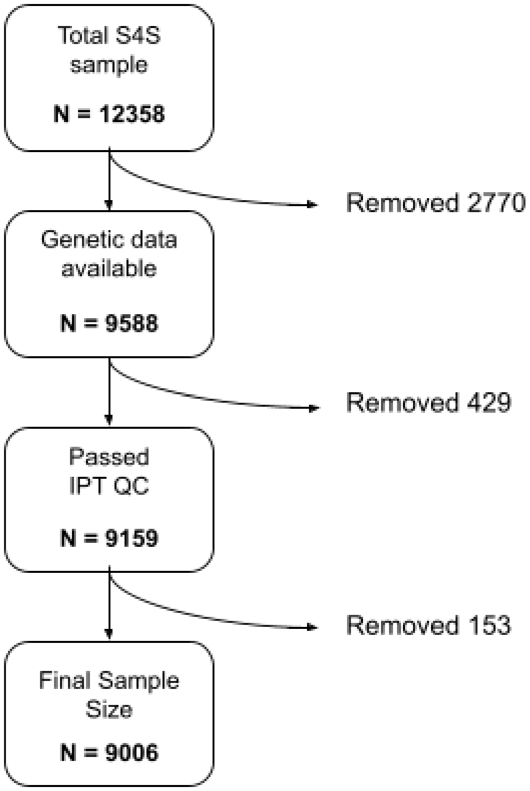

Supplemental Figure 1: Study Sample Size. Flow chart showing sample sizes at each quality control step.

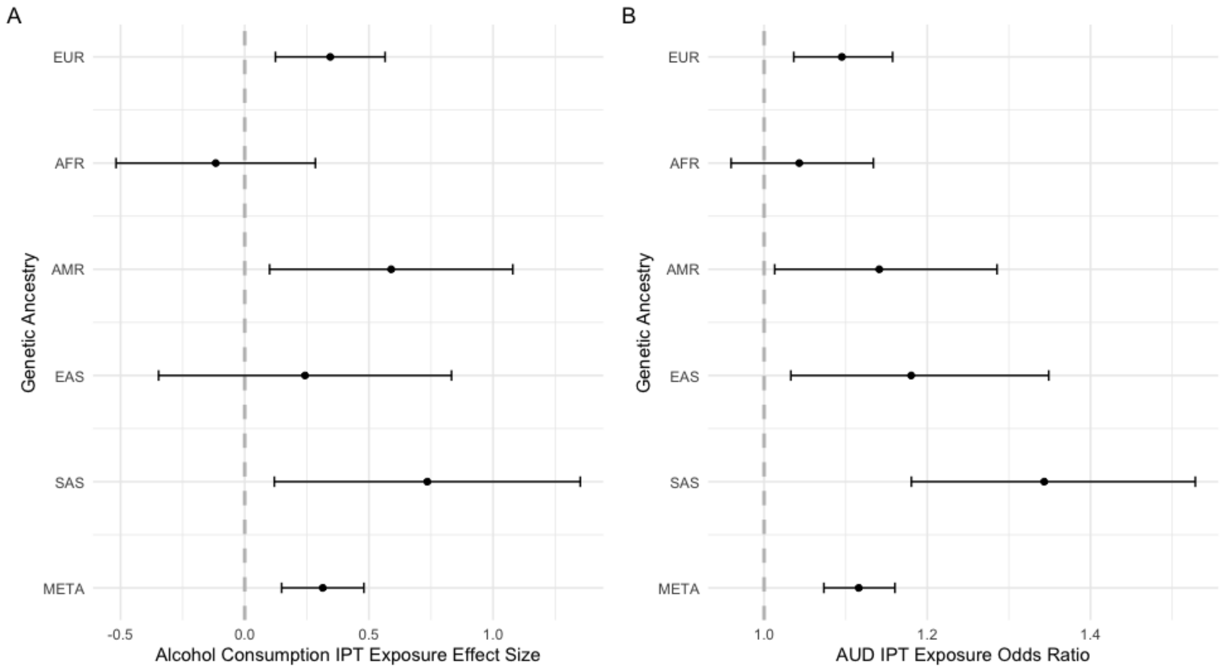

Supplemental Figure 2: Effect of IPT Exposure on Alcohol Behaviors Across Ancestries. Forest plots showing the interpersonal trauma (IPT) effect on A) alcohol consumption and B) alcohol use disorder (AUD) across ancestries and meta-analyzed (META).

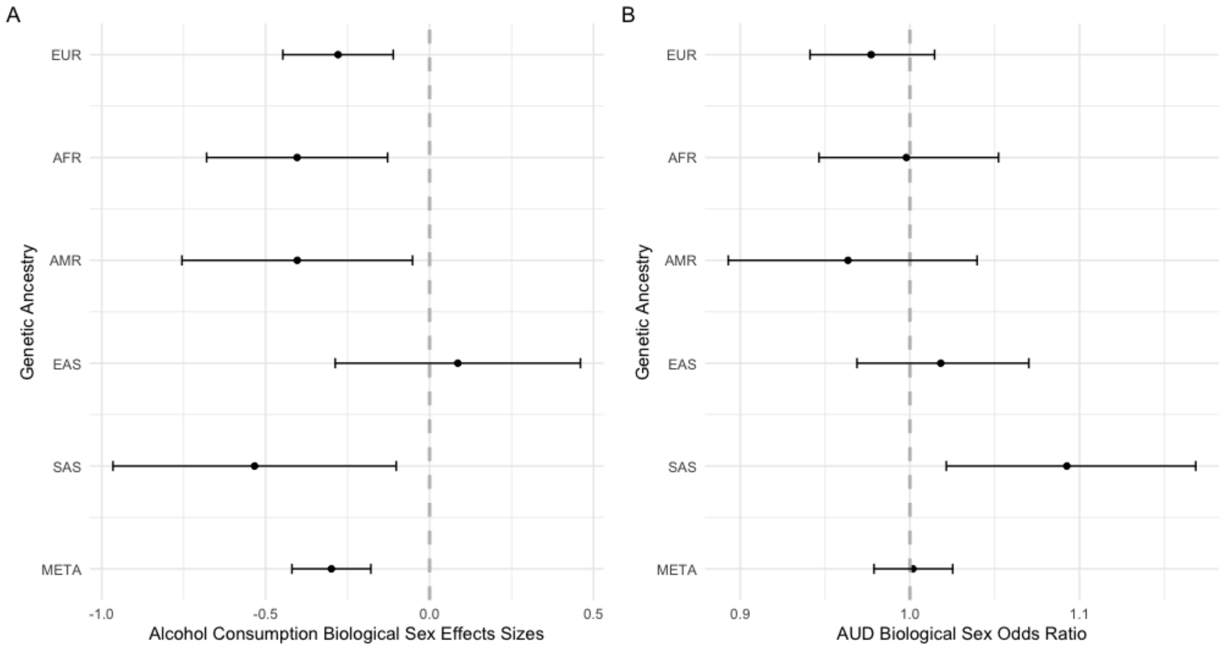

Supplemental Figure 3: Effect of Biological Sex on Alcohol Behaviors Across Ancestries. Forest plots showing the biological sex effect on A) alcohol consumption and B) alcohol use disorder (AUD) across ancestries and meta-analyzed (META).
